# Mucormycosis Following Tooth Extraction in a Diabetic Patient: A Case Report

**DOI:** 10.7759/cureus.9757

**Published:** 2020-08-15

**Authors:** Rajashri R, Muthusekhar M.R., Santhosh P Kumar

**Affiliations:** 1 Oral and Maxillofacial Surgery, Saveetha Dental College, Saveetha University, Chennai, IND; 2 Oral and Maxillofacial Surgery, Saveetha Dental College and Hospital, Chennai, IND

**Keywords:** mucormycosis, dental extraction, diabetes mellitus, immunocompromised, fungal infection, amphotericin b, rhino orbito cerebral, sinusitis, functional endoscopic sinus surgery, ct (computed tomography) imaging

## Abstract

Mucormycosis is a rare, opportunistic fungal infection, which can progress acutely or subacutely in immunosuppressed patients. It is a devastating disease in patients with poorly controlled diabetes mellitus and immunocompromised conditions. It causes decaying and extensive lesions of the soft tissue caused by fungi belonging to the order of Mucorales and is often fatal. Due to its aggressive nature, early detection and prompt management are of great importance for a good prognosis. We report a case of mucormycosis post-dental extraction in a patient with uncontrolled diabetes mellitus.

## Introduction

Mucormycosis is an opportunistic deep fungal infection, which ranks third after candidiasis and aspergillosis [1]. It is caused by an angioinvasive fungi in the order of Mucorales. They are nondemanding microorganisms growing at very variable temperatures. They are not very virulent, aerobic, and seen after two to five days of incubation on Sabouraud medium. Tissue necrosis due to invasion of blood vessels and subsequent thrombosis are the classic features of this disease, leading to rapid progression. This disease is rare representing 0.7% of invasive fungal infections that occur most commonly in immunocompromised people. Some 40%-50% of patients with mucormycosis are diabetic while other risk situations are IV drug addiction as well as immunosuppression induced by the treatment of coagulopathies after organ transplant [2]. However, the rare incidence of mucormycosis in apparently immunocompetent patients has also been documented [3-4]. They have a high mortality rate, and the key to success in treatment are early diagnosis, prompt administration of antifungals, and extensive surgical debridement.

## Case presentation

**Figure 1:**
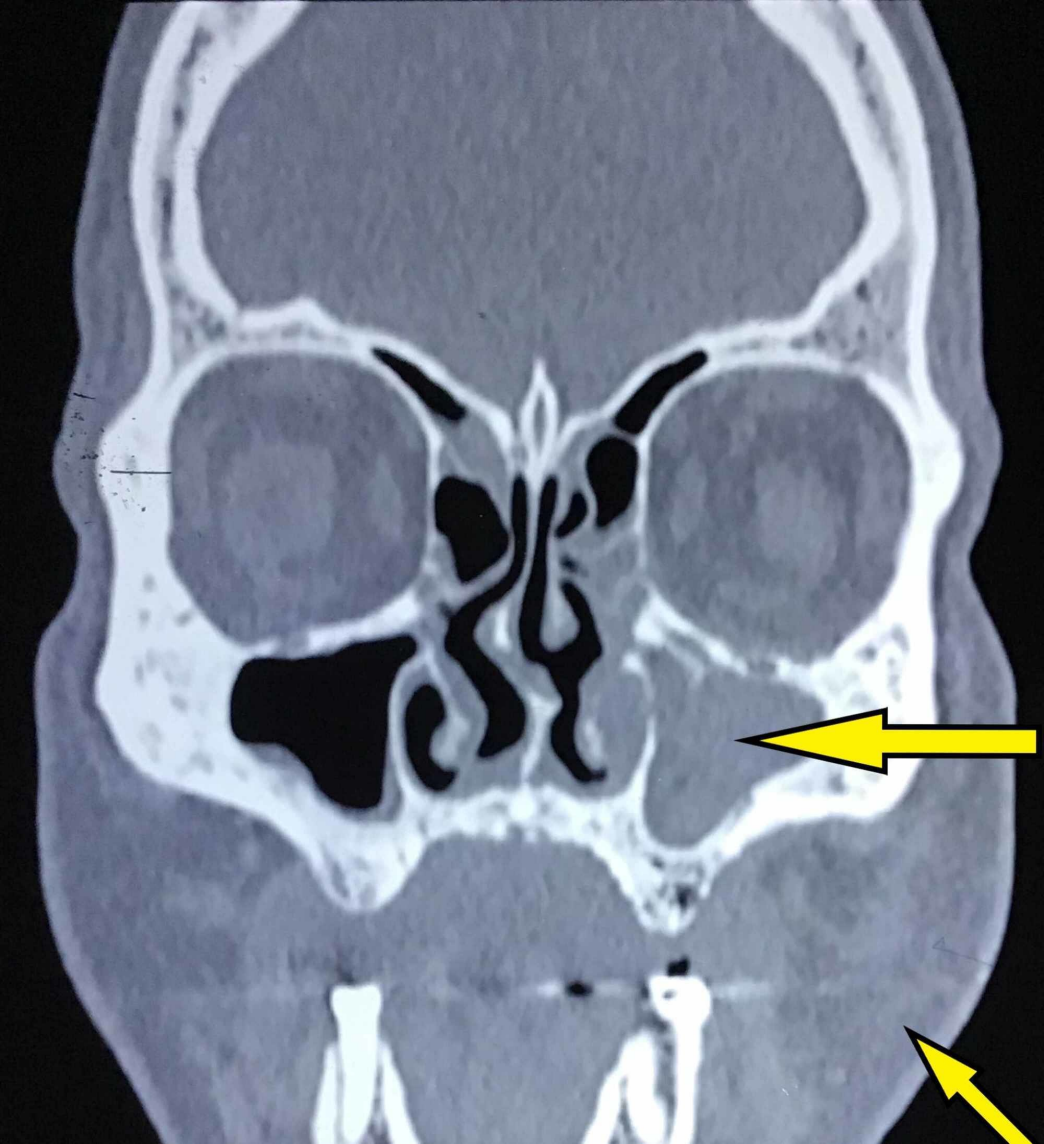
Preoperative facial CT showing facial swelling on the left side of face and obliteration of the left maxillary sinus.

A 55-year-old male presented to the Department of Oral and Maxillofacial Surgery at Saveetha Dental College and Hospital complaining of pain and sinusitis of his left alveolus following maxillary tooth extraction three weeks ago. Examination revealed facial cellulitis involving his left buccal, maxillary and infraorbital areas with difficulty in opening the mouth due to pain. Intra-orally foul-smelling, black discoloration of the mucosa of the left half of the palate with necrotic bone was observed. He was vitally stable and afebrile with a blood pressure 130/76 mmHg and a pulse rate of 82 beats/min; he was fully conscious with a Glasgow Coma Scale (GCS) 15/15, and gave a history of uncontrolled non-insulin-dependent diabetes mellitus. The patient was admitted in our department and an incisional biopsy taken from the left maxillary alveolar ridge revealed the presence of nonvital bone, necrotic fibrous connective tissue, foreign material, and flattened, broad, wide-angle hyphae with thrombosis suggestive of mucormycosis. Laboratory investigations revealed hemoglobin 12.5 g/dL (normal value: 12-16 g/dL), WBC count of 12,700 cells/cu.mm (normal value: 4,500-11,000 cells/cu.mm), differential count of 78% neutrophil (normal value: 55%-70%), 19% lymphocytes (normal value: 20%-40%), 2% eosinophil (normal value: 1%-4%), 1% monocyte (normal value: 2%-8%), platelet count of 3 lakh cells/cu.mm (normal value: 1.5-4.5 lakh cells/cu.mm), serum urea of 53 mg/dL (normal value: 5-20 mg/dL), serum creatinine of 1.2 mg/dL (normal value: 0.8-1.3 mg/dL), serum glutamic-oxaloacetic transaminase (SGOT) of 12 IU/L (normal value: 5-40 IU/L), and HBA1C of 14.4% (normal value: 4%-5.6%). Facial and brain MRI was performed which revealed mucolytic and destructive foci involving the left maxilla, posterior aspect of the left nasal bone, left pterygoids, greater wing of the sphenoid and left temporal bone and also large foci diffusing peripherally involving the left parapharyngeal space extending to the base of the skull and a peripheral ring-enhancing foci in the anterior temporal lobe with adjacent perifocal edema suggestive of possible intracranial extension. Facial CT showed obliteration of the left maxillary sinus and swelling on the left side of face (Figure 1).

**Figure 2:**
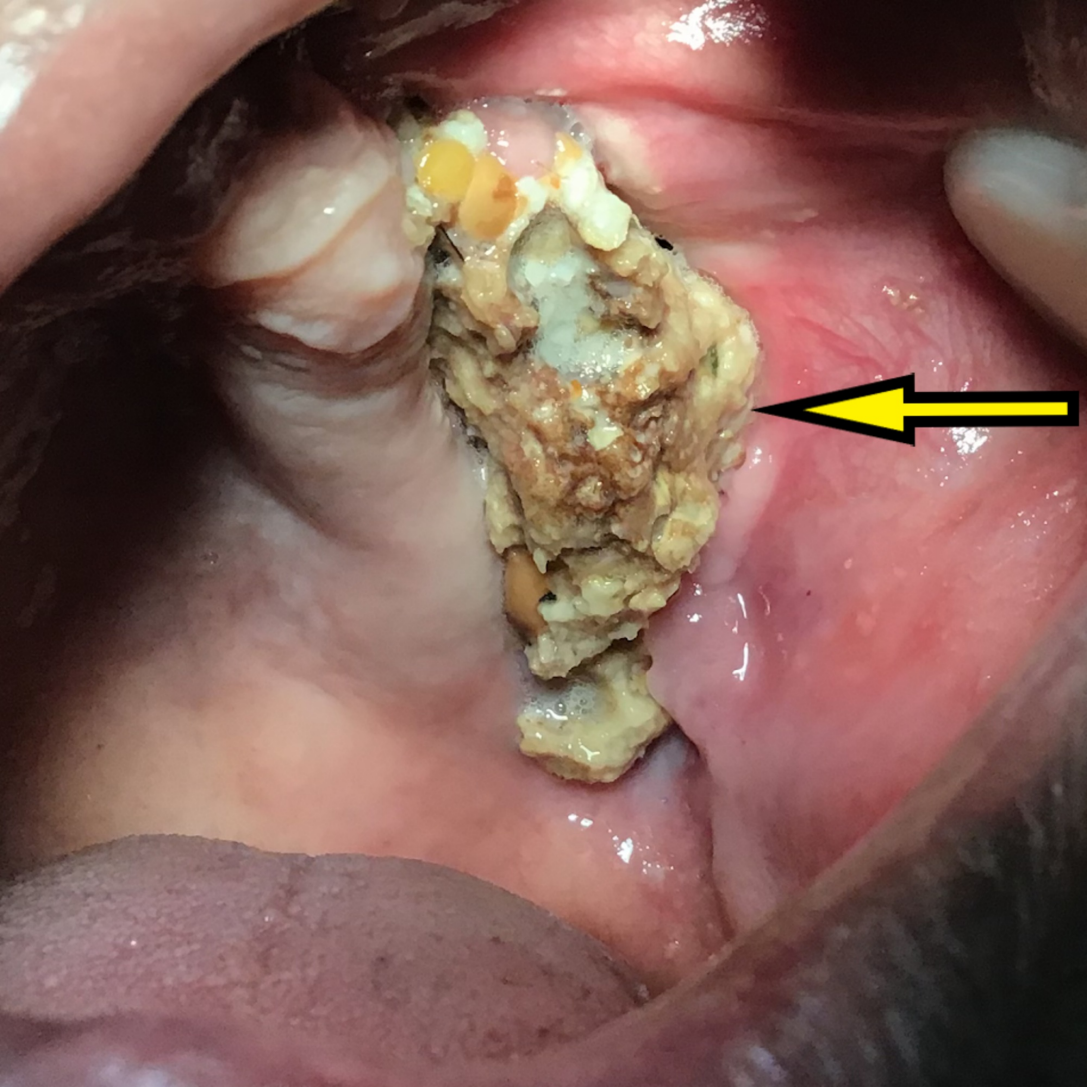
Intraoral view at 14 days showing healthy mucosa and necrosed alveolar bone.

Clinical and radiographic data were suggestive of early presentation of rhino-orbito-cerebral mucormycosis. Following confirmation of the diagnosis within four to five days, the patient was hospitalized and his blood sugar was controlled by the physician. Functional endoscopic sinus surgery (FESS) under general anesthesia was carried out for sequestrectomy of maxilla, total curettage of the maxillary antrum and complete debridement was done. The patient was administered IV amphotericin B as an infusion at 1 mg/kg/day in 100 mL of 5% dextrose over one to two hours for a period of 14 days with daily monitoring of kidney functions and electrolyte levels. The patient was discharged from the hospital after 14 days with an improved general condition. Healthy granulation tissue was seen and the wound healed uneventfully in 14 days (Figure 2).

**Figure 3:**
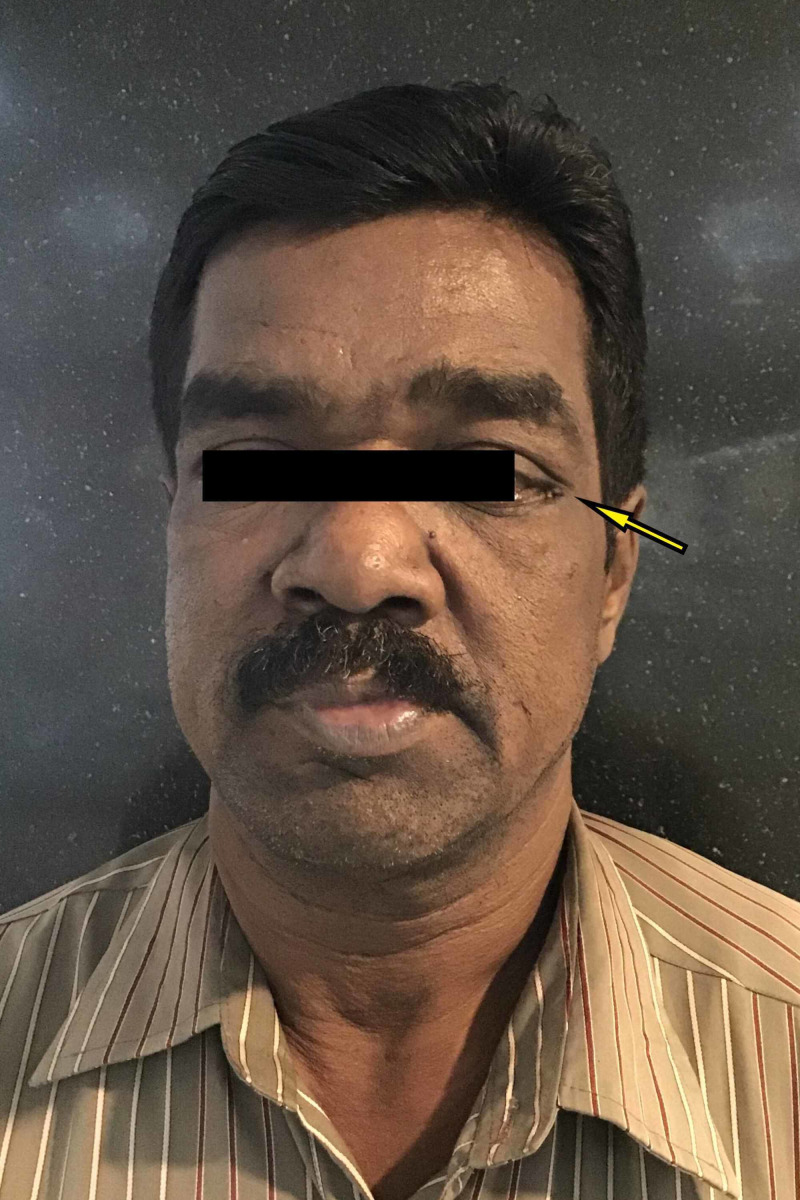
Postoperative clinical profile picture of the patient showing resolution of facial swelling and granulation of extraoral sinus near the lateral canthus of the left eye.

Resolution of facial swelling and granulation of extraoral sinus was observed at the end of 14 days (Figure 3).

**Figure 4:**
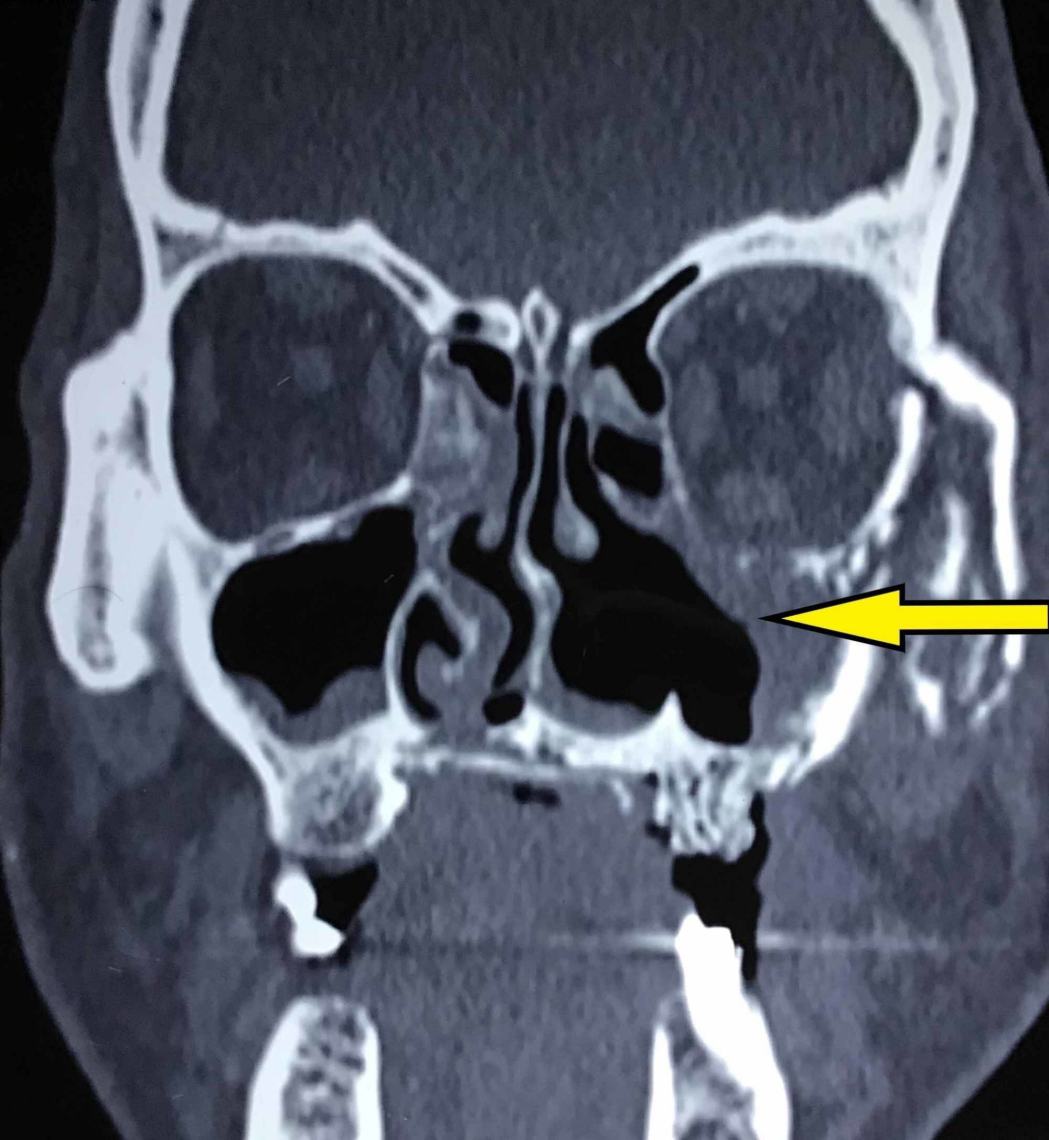
Postoperative CT at three months follow up revealing resolution of sinus obliteration following treatment.

The CT of facial bones at three months follow up revealed osteolysis of left maxilla, hard palate, posterior aspect of the nasal bone, left zygoma, left orbit, left sphenoid and bilateral pterygoid process and clivus with mild to moderate thickening in all paranasal sinuses and nasal cavities with occlusion of left ostiomeatal unit and bilateral turbinate hypertrophy (Figure 4). 

## Discussion

Given the serious and potentially fatal complication of tooth extraction, we reported a case of nonhealing extraction socket in a diabetic patient leading to mucormycosis. In approximately 50% of mucormycosis infections, diabetes mellitus was the predisposing factor. This could be due to the greater availability of glucose, lower pH, persistent reduction of blood flow, and subsequent decreased serum inhibitory activity against the pathogen and increased expression of some host receptors that mediate invasion of human epithelial cells causing abnormal phagocytosis and cell-mediated immune abnormalities [5]. The site of entry of microorganism determines the clinical presentation of mucormycosis and the organ systems involved. The rhinocerebral mucormycosis, first described by Paltauf in 1885, affected several organs involving the nose, paranasal sinuses, orbits, and central nervous system. Rhinocerebral localization is the most frequent, representing 40%-49% of mucormycosis with the other forms being cutaneous, gastrointestinal, pulmonary, and disseminated [6]. Oral mucormycosis is caused by inhalation of spores or direct contamination of open oral wounds. Oral mucormycosis affecting immunocompromised patients, mainly diabetes mellitus has been reported in the literature. However, those occurring after tooth extraction are rare [7-8]. The clinical presentation of rhinocerebral mucormycosis is not very specific. It is associated with varying degrees of headache, fever, rhinorrhea, orbital-facial cellulitis, ocular and neurological damage. The appearance of necrotic lesions in the form of pressure sores in the orbital-nasal region, the palate or the floor of the mouth should suggest the diagnosis [9-10]. The functional and vital prognosis for this condition is serious and the mortality rate remains as high as 80% [11]. Evolution depends essentially on the early onset of the assumption of responsibility for favorable recovery [12]. Despite timely actions, cases of death and unfavorable sequelae such as hemiparesis or palatal fistula have also been reported [13-14]. In routine maxillofacial practice, intra-oral exposed bone is diagnosed clinically as osteomyelitis but leads to a different picture based on microbiological and histopathological examination. It can occur due to bacterial osteomyelitis, herpes zoster, trauma, iatrogenic infections, or fungal infections, such as mucormycosis or aspergillosis. Therefore, a clinical suspicion requires confirmation by histopathological examination. Although MRI is presumed to be a better diagnostic tool for many clinical conditions; CT scan is considered better than MRI as it enables visualizing bone destruction. Moreover, CT scans are more cost-effective than MRI. Radiologically, opacification of the sinus is a classical feature of mucormycosis. CT scans of the maxilla and orbit show membrane or periosteal thickening and bony disruption in such cases [15]. Treatment is based on systemic administration of amphotericin B and local irrigation, along with surgical debridement of necrotic tissue and the control of diabetes levels [16-18].

## Conclusions

We infer that quick diagnosis is crucial in the treatment planning and anti-fungal treatment enables speedy recovery. Diabetes mellitus is the most common risk factor with other risk factors being immunocompromised patients with pre-existing malignancies and recipients of bone marrow transplants. Most patients with diabetes suffer from mucormycosis involving the paranasal sinuses in the form of a rhinocerebral mucormycosis involving both the sinus and orbit and communicating to the brain. In the past, the mortality rate of the rhinocerebral type was 88%, but recently the survival rate of rhinocerebral mucormycosis averages 21%-73% depending on the circumstances. Liposomal amphotericin B is the drug of choice. The outcome is more favorable in 70% of the patients when surgical therapy was combined with anti-fungal therapy and balancing the diabetic levels. Though the incidence of mucormycosis following a tooth extraction is extremely low, nevertheless when it occurs, may cause significant morbidity and mortality.
